# A data-centric investigation on the challenges of machine learning methods for bridging life cycle inventory data gaps

**DOI:** 10.1111/jiec.70022

**Published:** 2025-04-21

**Authors:** Bu Zhao, Jitong Jiang, Ming Xu, Qingshi Tu

**Affiliations:** 1 Department of Environmental and Sustainable Engineering, University at Albany, State University of New York, Albany, New York USA; 2 School for Statistics, University of Washington, Seattle, Washington USA; 3 School of Environment, Tsinghua University, Beijing, China; 4 Sustainable Bioeconomy Research Group, Department of Wood Science, The University of British Columbia, Vancouver, British Columbia Canada

**Keywords:** data centric, data gap, industrial ecology, life cycle inventory, machine learning, similarity based

## Abstract

Life cycle assessment (LCA) is a systematic approach to quantify the environmental impacts of a product system from its entire life cycle. Despite its wide use in assessing mature technologies, the inventory data gap has been a fundamental challenge that limits the application of LCA to emerging new processes. Machine learning (ML) methods are among the possible solutions that can mitigate these data gaps in an automated and scalable way. Nonetheless, the performance of existing ML methods is unstable which limits the trustworthiness and generalizability of the models. In this study, we conducted a data-centric investigation to delineate the causes of the unstable performance using a similarity-based ML framework based on Ecoinvent 3.1 unit process (UPR) database. We found that the pattern of imbalance in the data for method development, manifest by the substantial differences in (1) flow and process availability and (2) the order of magnitude of their values, is a major cause of the unstable performance. We also identified the causes due to the challenges with ML method development workflow, particularly, the steps of data preprocessing, and ML model training (e.g., randomness in train–test data splits). In addition, we also tested the proposed ML method on the U.S. Life Cycle Inventory Database, where we observed that the generalizability of the method was highly influenced by the database size of the application. To address these issues, we proposed that further research should focus on reducing the barriers in database integration such that both the size and balance of the data for ML method development can be improved.

**Supplementary Information:**

The online version of this article (doi:10.1111/jiec.70022) contains supplementary material, which is available to authorized users.

## INTRODUCTION

Life cycle assessment (LCA) is a systematic approach to quantify the environmental impacts of a product system from its entire life cycle. An LCA model consists of a foreground inventory dataset and an associated background inventory dataset. Currently, there is a remarkable challenge in translating the information of real-world activities (e.g., electricity use and waste treatment) into a foreground inventory dataset, particularly for emerging technologies, due to the constraints of information collection and processing. As a result, there exists a remarkable gap between (1) the rapid technological development in fields such as biochemicals and biomaterials and (2) the lack of high-quality data available for assessing their potential environmental impacts. This gap compromises the efforts to accurately evaluate and improve the sustainability of emerging technologies, particularly during their design phase. For example, deep eutectic solvents (DES) are widely explored in emerging technologies as a means to reduce energy consumption for lignocellulosic material manufacturing. The challenge of quantifying the environmental impacts of such emerging technology stems from the lack of life cycle models of most DES chemicals (e.g., choline chloride, p-toluenesulfonic acid) (Shiva et al., 2020).

Process simulation is a common method for estimating the energy consumption, material, and waste flows of the chemical manufacturing processes. Effective as it is, there is a high knowledge barrier (e.g., chemical engineering expertise and knowledge of facility-specific design) for applying the process simulation (Parvatker & Eckelman, 2019). Machine learning (ML) methods exhibit remarkable scalability and extensibility across diverse domains, allowing for their application to extract patterns and insights from complex data that might be challenging for human experts to discern (Zhu et al., 2023). Accordingly, an increasing number of studies have been conducted to investigate the feasibility of applying ML methods to estimate the missing inventory data and the results demonstrate the incredible potential of these methods to solve the data bottleneck issue (Dai et al., 2022; Khadem et al., 2022; Köck et al., 2023; Liao et al., 2020; Liu et al., 2023; Meron et al., 2020). However, most of these efforts primarily focus on demonstrating the efficacy of the proposed method by comparing the performance between various algorithms or other baseline models. Meanwhile, the efficacy of these methods is highly varying in different studies, which is contingent upon the composition of training data, the extent of missing information, and many other factors. This may significantly limit the trustworthiness and generalizability of the models and the corresponding results. While, so far, no study has conducted a systematic analysis and discussion of these issues from a data-centric perspective.

In this study, we conducted a data-centric investigation to discuss several fundamental challenges observed from training a typical ML framework for estimating the missing inventory data. Specifically, we focus on analyzing the complexities and limitations inherent in LCI datasets and how these factors influence the application and performance of ML models. As a showcase, we select a similarity-based method (details can be found in Section 2) that was previously developed as the baseline framework to demonstrate these issues (Hou et al., 2018). Based on the modeling process and the results, we formulate a data-centric discussion (i.e., the critical role of training data) on potential issues and strategies to improve the method, with a focus on the following aspects:

- Imbalanced data. In an LCI database, there is usually a significant imbalanced representation of different industrial sectors and flows in the databases, that is, one industry may have a significantly higher number of unit processes in a given database than other industries or one flow has a significantly higher appearance among all processes than other flows. Meanwhile, due to the different physical units, the magnitude of the values in the database could also vary in a wide range. This imbalance in data may pose significant challenges for data preprocessing and compromise the applicability and reliability of the prediction results, limiting their effectiveness across diverse real-world scenarios.
- Issues with model development. The techniques involved in the data preprocessing step of the conventional ML method development pipeline may not be applicable to the case of LCI data prediction, given that the inherent physical meaning of inventory data may be lost during the process. In addition, the randomness in generating train–test data splits may cause severe instability of model performance, since the datasets for model development in this case are typically smaller compared to other conventional ML tasks.
- Generalizability to other databases that are not used for developing the original models. Given the different database organization schemas among LCA databases, it is highly likely that an existing ML model needs to be retrained in order for it to perform well on another database. The retraining and performance evaluation efforts are rarely reported in the literature and hence, need to be investigated.

## METHODOLOGY

### Data preprocessing

In this study, we utilized the unit process (UPR) database from the default model of Ecoinvent 3.1 as the reference. Here, the UPR database is a matrix with columns representing unit processes (e.g., production of 1 kWh electricity) and rows representing either intermediate flows (i.e., inputs required by each unit process from other unit processes) or elementary flows (i.e., resources and emissions that are used or released in human and industrial activities, e.g., water consumption and CO_2_ emissions). Each element of the matrix indicates the amount of a particular type of intermediate or elementary flow (row) associated with the unitary output of a particular unit process (column), for example, 1.07 kg CO_2_ emissions per kWh electricity production in hard coal power plants. The dimension of the original UPR database is 13,201 rows by 11,332 columns. As the Ecoinvent database incorporates a geographical inheritance feature, lots of local processes are actually created as child of global parent processes (Weidema et al., 2013). These child processes inherit all associated flows from their parent processes by default, resulting in some local processes being generated as an exact copy of the global process with only uncertainty adjusted. Similarly, some of these processes are estimated based on the same proxy or attributed using the same weights, resulting in the exact same values for totally different processes. For example, the two processes of “waste brick//[CH] treatment of waste brick, recycling” and “waste cement-fiber slab//[CH] treatment of waste cement-fiber slab, collection for final disposal” have the exact same values. To maintain data integrity and prevent potential data leakage, we kept only one unique process (as the representative) by removing other duplicate processes (1463 processes in total). Additionally, we also removed the processes with the information of zero or only one flow (967 and 748 processes, respectively). After these procedures, we eliminated any empty rows (flows) or rows with only one element in the UPR matrix (1067 and 6892 flows, respectively). We repeated these procedures until no more processes and flows were removed. To remove the effects of the units on evaluating the distance (similarity), we conducted a normalization of the processed UPR matrix. Specifically, we conducted a row-wise min–max normalization (also known as min–max scaling) to rescale the flow values within a range between 0 and 1. As a result, the processed UPR matrix has 5242 intermediate and elementary flows (row) and 8154 processes (column). In the following experiments, we randomly split the UPR database into a training set (6154 processes, around 75% of the total processes, which is a typical ratio for regression tasks) and a test set (2000 processes, around 25%) to demonstrate the performance of the trained model.

### Model pipeline

In this study, a similarity-based link prediction model was used as the reference model to estimate missing unit process data using limited available information (Hou et al., 2018). This approach is a technique from network science to predict missing links based on existing network structures, which is frequently used in recommendation systems and social network analysis. The approach was selected due to its representativeness, general applicability, and efficacy demonstrated in the previous studies (Hou et al., 2018). Here, we treated the UPR database as a technology network and randomly assigned a certain proportion (5% in this study) of missing values to part of these processes. We selected this missing proportion based on the findings from our previous study (Hou et al., 2018), which indicates that missing more than 10% of data can notably compromise the performance of the model. In our modeling framework, we treated these processes with missing values as some new processes with partial information and applied this method to estimate these missing data by solely using the information from the remaining processes. The intuition of this method assumes that similar processes should also have similar inputs required or output generated (i.e., similar flows) (Hou et al., 2018; Zhao et al., 2021), and a simple version of this method, that is, proxy method, has been widely used by LCA practitioners when the primary information is not available. For any new unit process with part of its data missing, we could use the information of its similar processes in our UPR database to help predict its missing part.

The similarity-based link prediction model consists of two steps, the first is to calculate the distance-based similarities between this new process with all the known processes in the UPR database based on the non-missing parts (Zargar et al., 2022). After that, the model estimates the missing parts using the *k* nearest neighbors (kNN) method, which uses the weighted mean (using similarity as the weights) of the *k* most similar processes as a prediction for the missing part. The detailed procedure is defined as follows:

1. Similarity calculation

We first computed the Minkowski distance of process *j* in the test set with other processes in the training set based on the non-missing parts which are defined as (Chomboon et al., 2015):

1 $${d_{ij}} = {\left( {\sum_{t\in{S^{{\mathrm{known}}}}} {{\left| {{\alpha _{ti}} - {\alpha _{tj}}} \right|}^q}} \right)^{\frac{1}{q}}}$$

where $${\alpha _{ti}}$$ is the value of flow *t* in the training process *i*, $${\alpha _{tj}}$$ is the value of flow *t* in the test process *j*, and *q* is the order or degree of the distance metric for the Minkowski distance. $$S, \;{S^{{\mathrm{known}}}}, \;and$$$${S^{{\mathrm{missing}}}}$$ represent the index for all flows, the index for the know flows, and the index for the missing flows which are defined as below:

2 $$ \def\eqcellsep{\;}\begin{array}{@{}*{1}{c}@{}} {S\; = \left\{ {{\mathrm{the\;total\;flows\;}}\left( {{\mathrm{intermediate\;and\;elementary}}} \right){\mathrm{in\;the\;process}}} \right\}}\\ { = \left\{ {{\mathrm{known\;flows\;}}\left( {{\mathrm{intermediate\;and\;elementary}}} \right){\mathrm{in\;the\;process}}} \right\} \cup \{{\mathrm{missing\;flows}}\;\left( {{\mathrm{intermediate\;and\;elementary}}} \right){\mathrm{in\;the\;process}}}\}\\ { = {S^{{\mathrm{known}}}} \cup {S^{{\mathrm{missing}}}}, where\;{S^{{\mathrm{known}}}} \cap \;{S^{{\mathrm{missing}}}} = \emptyset} \end{array} $$

where the number of flows for each set can be represented as $| S | = m$, $| {{S^{{\mathrm{known}}}}} | = m - p$, $| {{S^{{\mathrm{missing}}}}} | = p$.

Then, the similarity of process *j* and process *i*, ${s_{ij}}$, is then calculated based on their distance ${d_{ij}}$.

2 $${s_{ij}} = \frac{1}{{{d_{ij}} + 1}} $$

2. Missing data estimation

Each missing data point in the test set process *j* is estimated by averaging the corresponding data in the *k* most similar processes weighted by their similarities, which are calculated by:

3 $${e_{tj}} = \frac{{\sum_{i = 1}^k \left( {{\alpha _{ti}}{s_{ij}}} \right)}}{{\sum_{i = 1}^k {s_{ij}}}}\;$$

where *k* represents the number of most similar processes in the training set used to estimate the missing data in the test set and ${\alpha _{ti}}$ is the corresponding flow *t* of the *i*th similar process when the training processes are ranked in descending order of similarity. More details of this method could be found in Hou et al. (2018).

During the training, we found the best *q* and *k* combination which achieved the lowest estimation errors for each training dataset. The final performance of the proposed model was evaluated on the test set and through the leave-one-out cross-validation (LOOCV), that is, the model is trained on all processes in the training set except one and validated by each of these processes through the entire dataset (Wong, 2015). All data processing and experiments were conducted using the R2022a software.

### Evaluation metrics

We used mean absolute percentage error (MAPE) to select the best model structure and evaluate the performance of the selected model with different numbers of data missing (de Myttenaere et al., 2016).

4 $${\mathrm{MAPE\;}} = \frac{1}{{\left| {{S^{{\mathrm{missing}}}}} \right|}}\sum_{t\in{S^{{\mathrm{missing}}}}} \left| {\frac{{{\alpha _{tj}} - {e_{tj}}}}{{{\alpha _{tj}}}}} \right| = \frac{1}{p} \sum_{t\in{S^{{\mathrm{missing}}}}} \left| {\frac{{{\alpha _{tj}} - {e_{tj}}}}{{{\alpha _{tj}}}}} \right|{\mathrm{\;\;}}$$

where ${\alpha _{tj}}$ is the value of flow *t* in the test process *j*, ${e_{tj}}$ is the estimation of flow *t* in the test process *j*.

MAPE is a metric widely used to measure the accuracy of predictions in comparison to actual observed values, which offers insight into the inherent bias or inclination of a forecasting approach to either overestimate or underestimate the real values. In our experiments, each process with missing data generated a corresponding MAPE. When assessing the overall model performance across the entire dataset, we chose to calculate the median of the MAPE from all processes as the final evaluation metric. Specifically, due to the huge difference (up to 10^12^) in the order of magnitude for different flows in different processes, we chose to use the median MAPE for each process as the primary evaluation metric, rather than some other commonly used metric like coefficient of determination (*R*^2^), to avoid the model performance being dominated by a few large values.

## RESULTS

### Baseline model performance

In this study, we evaluated the baseline model performance under the situation with 5% of the missing data (as described in the model pipeline section). Specifically, we conducted two rounds of grid searches to find the optimal model structure (Figure 1 of Supporting Information S1). For the first round, the *q* is set in the range of 0.001 to 10 (for the Minkowski distance calculation) and *k* from 1 to 20 (the number of nearest neighbors). The optimal model structure is *q* = 0.1 and *k* = 2 (Figure 1a of Supporting Information S1). We then zoomed in and refined the range for both hyperparameters and conducted the second-round grid search where *q* is from 0.001 to 0.2 and *k* from 1 to 5. Based on the results of LOOCV (Figure 1b of Supporting Information S1), the optimal hyperparameters were found to be *q* = 0.075 and *k* = 2. For the prediction of the non-zero missing flows (i.e., those flows existent in the process of interest), the model achieved an MAPE of 34.68% during LOOCV and 35.41% on the test set, indicating the ideal and robust performance of the model. While, for the zero flows (i.e., those non-existent flows in the process of interest), the misclassification errors (predict the zero flows with non-zero values) for the model are extremely low with 0.15% during LOOCV and 0.16% on the test set, indicating that the model has a very low probability of generating erroneous values for non-existing flows. All these results suggest that the model could accurately identify which flows really exist in the target process (i.e., the new process with missing data) and provide reasonable estimations of their values in the meantime.

When we take a closer look at how the model works, the distance parameter *q* (0.075) is quite small. With such a small *q*, the distance measure becomes extremely sensitive to small differences between different processes which enables the finding of trivial differences in the normalized data. Meanwhile, the small value of *k* (i.e., 2) demonstrates that by only utilizing information from just a few of the most similar processes is sufficient to provide reasonable estimations for the target process. This result not only demonstrates the model's effectiveness but also validates the rationality of the proxy method widely employed in real-world LCI practices, using closely related data to estimate unknown values. To further justify these points, we selected 10 representative unit processes as case studies to demonstrate the model's effectiveness. For each unit process, we identified its most similar processes and calculated the MAPE of the estimation. As presented in Table Table 1, the model successfully identified relevant similar processes and generated reasonable estimations for the missing flows in the processes of interest. For example, for the unit process “petrol, unleaded//[CH] petroleum refinery operation,” the most similar processes are “diesel//[CH] petroleum refinery operation” and “naphtha//[CH] petroleum refinery operation,” which are highly related processes in the same industry and same region and the MAPE is only 0.513%. Surprisingly, the model also identified some processes that are not intuitively similar, such as the most similar processes for “white spirit//[RER] white spirit production” are “lubricating oil//[RER] lubricating oil production” and “alkylketene dimer sizing agent, for paper production//[RER] alkylketene dimer sizing agent production, for paper production” which are processes in totally different industries. This suggests that the similarity-based method can enhance proxy selection in cases where traditional domain knowledge is limited. Unlike domain knowledge, which typically only identifies similar processes within the same industry, our similarity-based approach can identify similar processes across different industries by analyzing the structure and values of the target process. This capability broadens the scope of conventional estimation methods, enabling the discovery of analogs beyond the constraints of industry classification. When we take a step further, as shown in Table 1 of Supporting Information S1, the estimated flow values are highly aligned with the true values of the missing flows (after preprocessing). These examples demonstrate the model's capability to leverage similarities between processes for accurate data estimation. Specifically, the model demonstrates a balanced performance regardless of the category or magnitude of the flows, which may be due to the normalization conducted during data preprocessing.

**TABLE 1:** Examples of processes of interest, their respective similar processes, and the mean absolute percentage error of the estimation.

Process name	First similar process	Second similar process	MAPE (100%)
**Copper sulfate//[GLO] copper sulfate production**	Nickel sulfate//[GLO] nickel sulfate production	Sodium arsenide//[GLO] sodium arsenide production from Imaperial smelting furnace	4.19E-15
**Sulfur dichloride//[RER] sulfur dichloride production**	Thionyl chloride//[RER] thionyl chloride production	Sulfur dichloride//[RoW] sulfur dichloride production	1.22E-14
**White spirit//[RER] white spirit production**	Lubricating oil//[RER] lubricating oil production	Alkylketene dimer sizing agent, for paper production//[RER] alkylketene dimer sizing agent production, for paper production	1.38E-14
**Petrol, unleaded//[CH] petroleum refinery operation**	Diesel//[CH] petroleum refinery operation	Naphtha//[CH] petroleum refinery operation	5.13E-01
**Dimethylamine//[RER] dimethylamine production**	Methylamine//[RER] methylamine production	Dimethyl ether//[RER] dimethyl ether production	5.37E-01
**Chlorine, gaseous//[RoW] sodium chloride electrolysis**	Iron sulfate//[RoW] iron sulfate production	Protein feed, 100% crude//[RoW] soybean meal and crude oil production, mechanical extraction	1.46E+00
**3-Methyl-1-butyl acetate//[RoW] 3-methyl-1-butyl acetate production**	Butyl acetate//[RoW] butyl acetate production	Isopropyl acetate//[RoW] isopropyl acetate production	3.24E+00
**Aluminium sulfate, powder//[RoW] aluminium sulfate production, powder**	Iron sulfate//[RoW] iron sulfate production	Polymer foaming//[RoW] polymer foaming	7.86E+00
**Ethylene, average//[RoW] ethylene production, average**	Propylene//[RER] propylene production	Butene, mixed//[RoW] butene production, mixed	1.33E+01
**Limestone, crushed, for mill//[RoW] limestone production, crushed, for mill**	Limestone, crushed, washed//[RoW] limestone production, crushed, washed	Limestone, crushed, for mill//[CH] limestone production, crushed, for mill	2.02E+01

### The challenges related to the special characteristics of UPR database

Although the results demonstrate the great potential of the baseline model for estimating the missing flows in the target processes, there are still multiple challenges due to the uniqueness of the UPR dataset. In this section, we discuss the challenges we have faced during the data preparation.

#### Data sparsity and imbalance

Because the UPR database only includes on-site energy and resource use and emission data for each process, most entries in the UPR matrix are zeros. Thus, the Ecoinvent UPR database is a sparse matrix, in which only 0.51% (1.41% for the preprocessed dataset) of entries are non-zero. This means, for a typical process, it usually only includes information about 70 flows, with the remaining flows as zeros, and any missing of these non-zero flows will result in significant information loss and bring great difficulties to the future estimation task.

**FIGURE 1:**
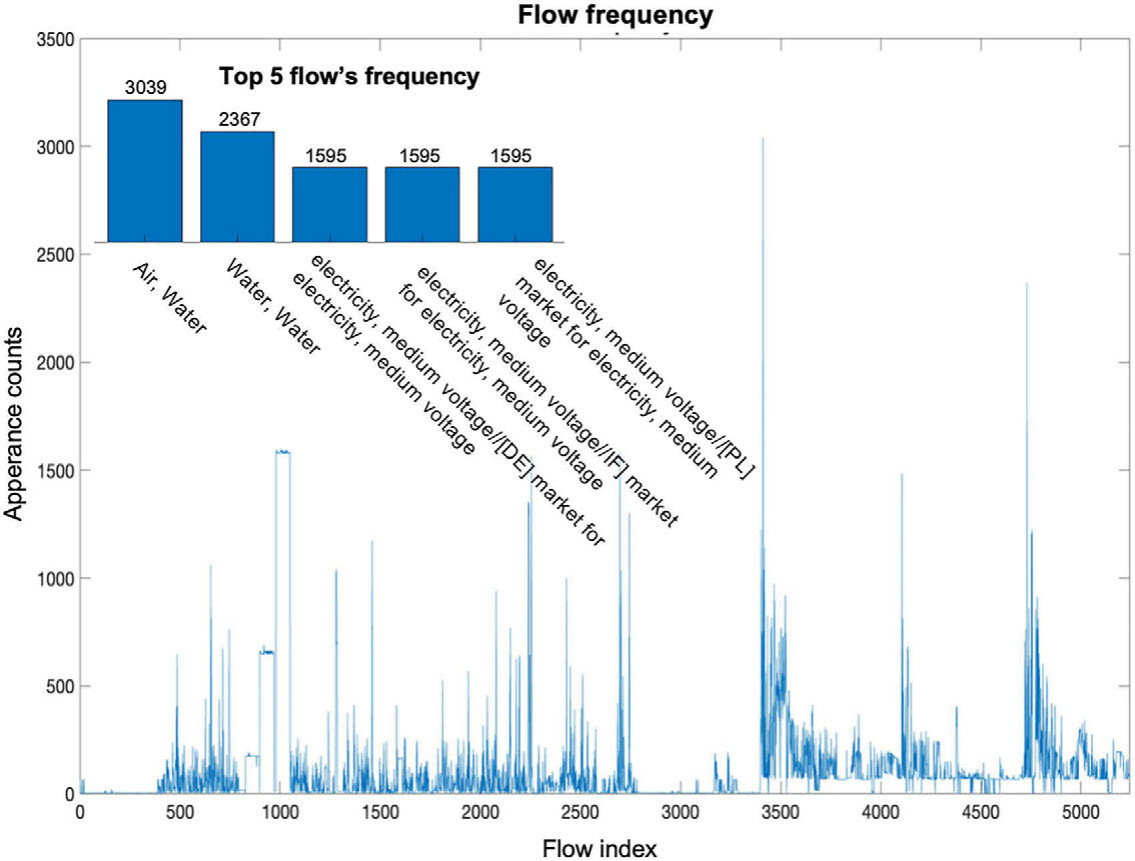
Imbalance in flow appearances. Flows are arranged in order with their row indexes appearing in the UPR matrix. The plot shows the frequencies of each flow that appear in all processes. The subgraph on the upper right represents the top five flows and their frequencies (the values above the bar) among all flows. The first two flows represent the emission of water to a specific “compartment” of the environment (e.g., water body and air) and the other three flows are the medium voltage electricity usage from different geographic locations (e.g., DE, IF, and PL). Underlying data for this figure are available in Sheet Data_for_Figure1 of Supporting Information S1.

**FIGURE 2:**
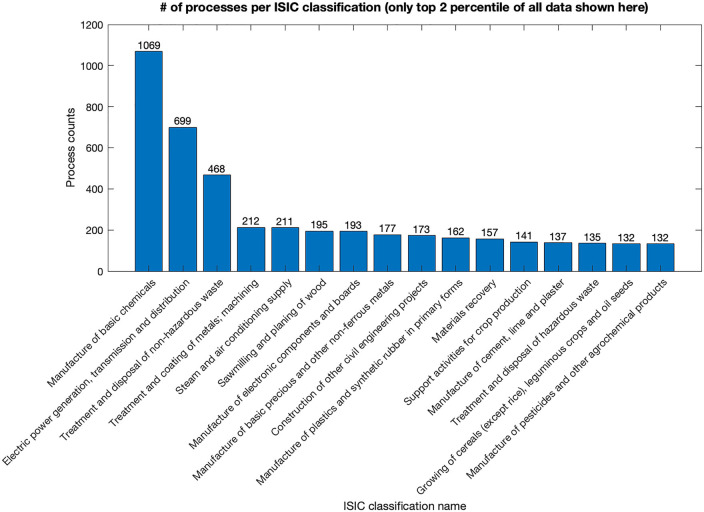
Imbalance in process appearance in different ISIC classifications. Each process has an ISIC classification name to which it belongs. The number of each ISIC classification, that is, process count, is counted and sorted in descending order. This figure shows only the ISIC classification with process count in the top 2 percentile. Underlying data for this figure are available in Sheet Data_for_Figure2 of Supporting Information S1.

Meanwhile, sparsity often leads to imbalanced datasets where some classes or categories are underrepresented. These imbalance issues manifest in multiple aspects. In the preprocessed UPR database, we can find a clear imbalance distribution of different flows. As shown in Figure 1, the appearance of both intermediate and elementary flows varies substantially throughout the entire UPR database. The top 20% of flows with the highest appearance accounted for 72% of the total non-zero flows. These dominant flows, such as water and electricity, are usually some basic flows that exist for almost every process, which is consistent with our expectations. Also, the number of unit processes under each International Standard Industrial Classification (ISIC) could vary from 1069 (“Manufacture of basic chemicals” in Figure 2) to 1 (Silviculture and other forestry activities). This can lead to biased predictions and poor performance for those minority classes.

#### Data magnitudes and normalization

**FIGURE 3:**
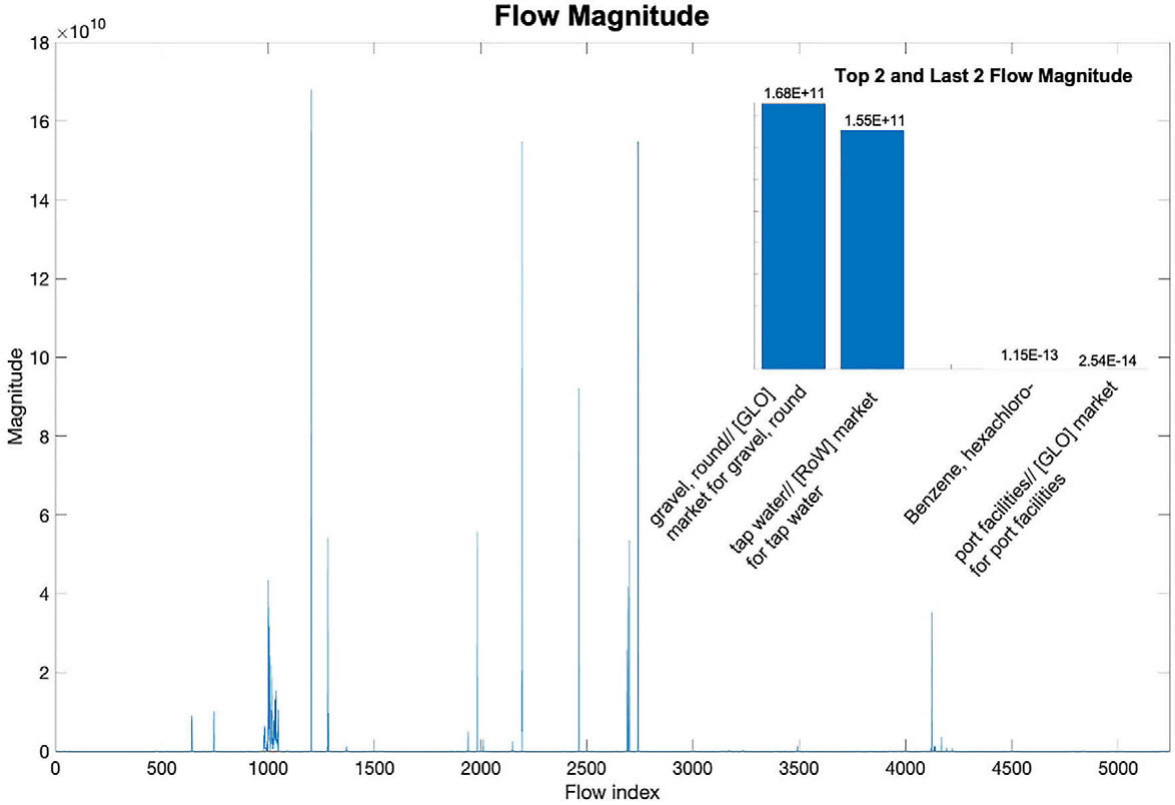
Magnitude differences between flows. Flows are arranged in order with their flow indexes and the magnitude of each flow is the largest value of each flow. The specific values of the two largest magnitudes and the two smallest non-zero magnitudes, with their corresponding flow names to the flow index are displayed in the upper right corner. Underlying data for this figure are available in Sheet Data_for_Figure3 of Supporting Information S1.

Unlike traditional matrices, the UPR database represents the underlying technology network which has clear physical and chemical meanings. Considering the different units of processes/flows and the inherent differences in industrial activities, the order of magnitudes of different elements in the database may vary greatly. In our original UPR database without preprocessing (Figure 3), the largest value was 1.68 × 10^11^ (“gravel, round//[GLO] market for gravel, round”/kg) found in the unit process of “hydropower plant, reservoir//[CA-QC] hydropower plant construction, reservoir”/unit. On the other hand, the smallest non-zero value was only 1.83 × 10^−21^ (“Benzene, hexachloro-”/kg) found in the unit process of “feldspar//[RER] feldspar production”/kg. In addition, even in a single process or flow, the difference in the order of magnitude could be remarkable. For example, for the flow “Occupation, industrial area”/m^2^ × year, the values ranged from 5 × 10^−5^ to 5 × 10^7^ in different processes. The varying orders of magnitude may naturally assign higher weights to those larger values and skew the performance of the trained model towards a certain range of values.

To remove the influence of different units and vast differences for the magnitudes of different flows, we conducted a min–max normalization of the processed UPR matrix to ensure that features are on a consistent scale (i.e., 0–1). This process prevents features with larger values from dominating the learning process and helps algorithms converge faster during optimization. Additionally, this process eliminates the effects of unit selection during distance calculation. For example, if the unit of a process changes from “kg” to “ton,” the underlying information remains the same, but without normalization, the distance calculation would be affected. Normalization ensures that such unit changes do not impact the similarity assessment between processes. Despite this, this process may obscure the physical meaning of the dataset and make the distance calculation hard to interpret. Meanwhile, considering the remarkable differences regarding the order of magnitudes within each flow, the min–max scaling could only have limited effects on mitigating differences in orders of magnitude and was unable to completely solve the problem of the skewed distribution of the data. As shown in Figure 2 of Supporting Information S1, after conducting min–max scaling for each flow, the median non-zero values for each flow still vary from 5.21 × 10^−18^ to 1, indicating the intrinsic differences among different flows.

To totally remove the magnitude differences for each flow, the log transformation seems to be a potential solution. However, the great number of zeros in the dataset could cause challenges for using log transformation. A typical approach to solve this problem is to add a constant (usually a very small value or one) to every element in the dataset. However, adding a very small value will introduce redundant information that may change the physical meaning of the data after the log transformation (i.e., making those non-existent flows, which originally have zero values, non-zero hence artificially including them in the calculation), which may mislead the calculation in the distance-based method. On the other hand, adding the value of one would avoid the redundant information issue, as the zero value still maintains zero after transformation (i.e., log (0 + 1) = 0) but still fails to alleviate the magnitude difference issue. Compared with that, the Box–Cox transformation is a more flexible and versatile method, which is designed to address a wide range of data distributions by introducing a parameter λ (log transformation is a specific case when λ is set to 0). However, it is important to note that the Box–Cox transformation is typically applied to data represented in vector form, where the mathematical properties align with the method's assumptions. Recent studies have demonstrated the applicability of Box–Cox transformation on two-dimensional data (like matrix or images) (Cheddad, 2020). Unfortunately, in our specific context, the inherent sparsity of our UPR database and the fact that the data lacked an upper bound, unlike the RGB values (i.e., a color value represents red, green, and blue light sources with values ranging from 0 to 255) of the image data, made it difficult to achieve a meaningful transformation outcome that would have enhanced our analysis.

### The challenges related to the model development

In addition to the issues with the UPR dataset, there are also certain issues which directly related to the model development. In this section, we discuss the challenges we have faced during the model construction.

#### Variation of model performance due to the randomness of train–test data splits

When we use random seeds to split the dataset into different training and test sets (i.e., the composition of the training and test sets changed), the performance of the model based on median MAPE can vary. This variation depends on how the data is divided into these subsets, which is a natural and inherent aspect of every ML experiment. This variability arises from the randomness of the data split and could have a notable impact on the outcomes of ML experiments and the reliability of model evaluations.

**FIGURE 4:**
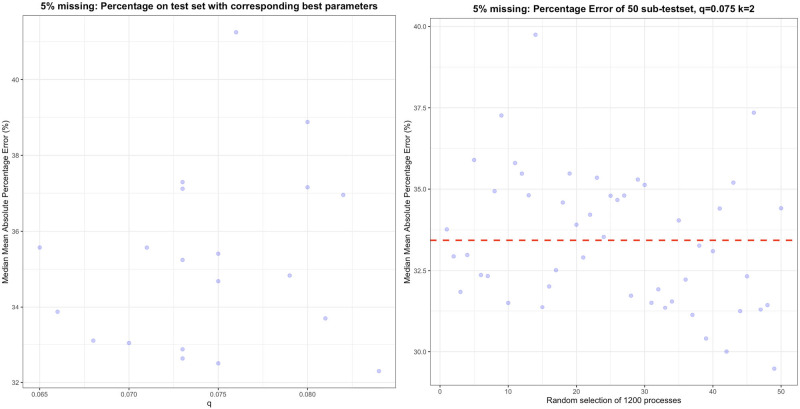
Variation of model performance due to the randomness of train–test data splits under the situation with 5% of missing data. In the left figure (a), we randomly chose training sets using 20 different seeds and used the respective optimal combinations of parameters (*q*, *k*) on their corresponding test sets to check the model performance. In the right figure (b), based on one fixed test set, we randomly selected subsets (1200 flows) from it 50 times using different seeds and showed the model performance on the respective sub-test sets using median mean absolute percentage error. The red dashed line represents the mean model performance. Underlying data for this figure are available in Sheets Data_for_Figure4(a) and Data_for_Figure4(b) of Supporting Information S1.

To demonstrate this point, we randomly split the UPR database into 20 different combinations of training sets (6154 processes) and test sets (2000 processes) by using 20 different seeds, that is, we created 20 different sets of training and test set combinations. As shown in Figure 4a, the optimal model structure could vary with the *q* ranging from 0.065 to 0.084, and *k* from 1 to 2. These models also showed quite different performances on the test sets and the best result was 25.82% better than that of the worst model. Meanwhile, even when we fixed the training set and determined the optimal model hyperparameters, the model performance still could vary when facing different test sets. As shown in Figure 4b, when we randomly extracted different subsets (1200 out of 2000) from the test set for 50 times, the lowest MAPE was 25.82% lower than the highest one. This means that for different runs of the same experiment, individual cases may not be a good representation of the overall model performance, leading to either overly optimistic or pessimistic estimations.

#### Variation of model performance due to the imbalance of data

**FIGURE 5:**
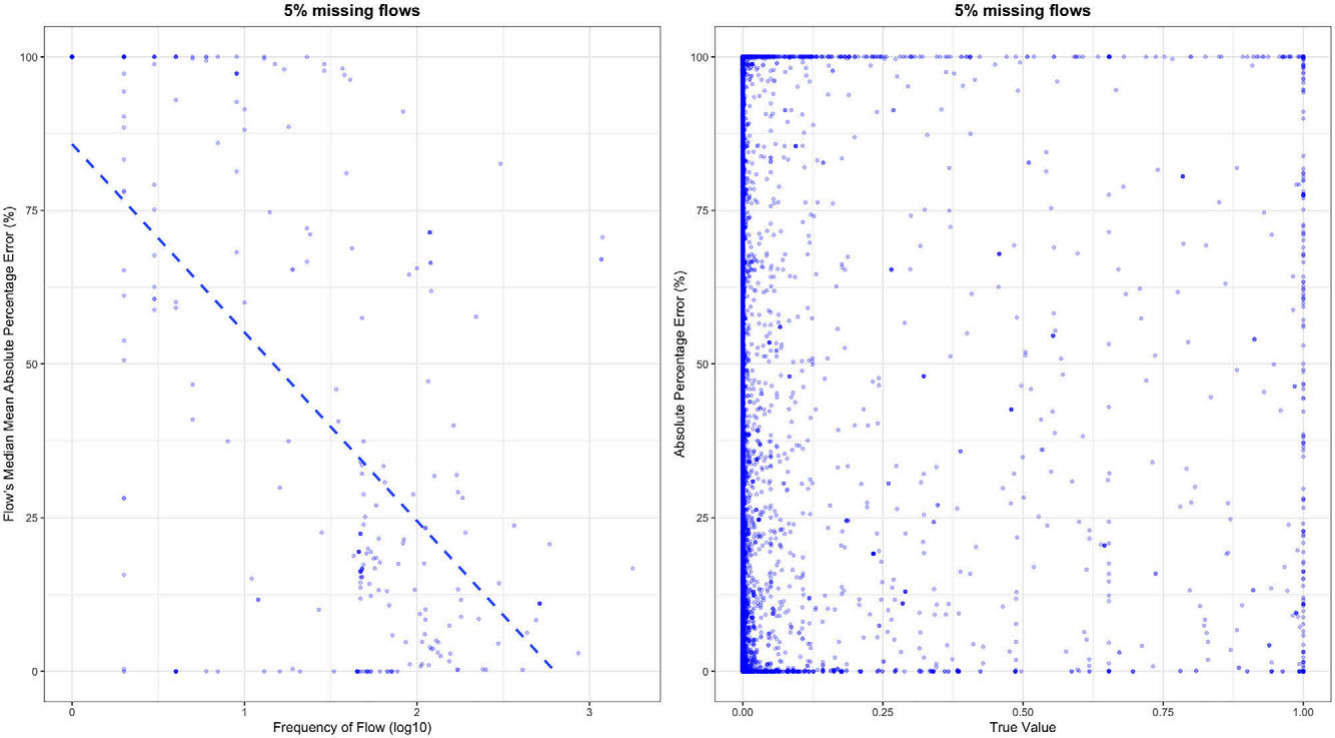
Variation of model performance due to the imbalance of data under the situation with 5% of missing data. (a) The scatter plot of the flow's median mean absolute percentage error under different flow frequencies. The trend line is represented by the blue dashed line. (b) The scatter plot of the percentage error under different value magnitudes. Underlying data for this figure are available in Sheets Data_for_Figure5(a) and Data_for_Figure5(b) of Supporting Information S1.

We conducted several experiments to explore the impact of the data imbalance on the prediction results. As shown in Figure 5a, the frequency of flow had a positive impact on the model performance. Flows with higher appearance frequencies tended to have a lower median MAPE on the prediction results. This trend can be attributed to the fact that for those flows with higher appearance frequencies, it is easier to find processes with similar flows as references. Whereas, for those flows rarely found in any process, the model struggles to discern patterns and find similar processes for comparison. This scarcity of reference data makes it challenging for the model to accurately predict values for these infrequent flows.

Regarding the data magnitude issue, we did not find a clear relationship between the magnitude of the flow and its MAPE (Figure 5b). This is mainly due to the effects of normalization which limit the values of different flows in a fixed range. Although this process has balanced the influence of different variables (flows), the physical meaning of the values has also been lost during this process. This may also be an issue for other ML models, such as artificial neural network, which needs to consider the trade-off between the model performance with the interpretability.

#### Model generalizability on a different database

To test its generalizability, we applied our methodology to another widely used UPR database, that is, U.S. Life Cycle Inventory (USLCI) Database. Compared with Ecoinvent, USLCI only provides national UPR information with fewer flows and processes (the dimension of the USLCI is 4074 rows by 638 columns). Similar to Ecoinvent, USLCI is also a sparse matrix with only 1.46% of its value as non-zero. After the same preprocessing procedure, the dimension of the USLCI is down to 3316 rows by 624 columns (1.8% non-zero). We then trained and tested the model performance on the preprocessed USLCI dataset using the same modeling pipeline. As shown in the heatmap (Figure 3 of Supporting Information S1), this similarity-based link prediction model has worse performance compared with the results based on the Ecoinvent. The optimal model structure (*q* = 0.1 and *k* = 2) achieved an MAPE of 50.6% during LOOCV, which is about 46% higher than the MAPE for Ecoinvent. The reason for such a disparity in the model performance between the two databases was mainly due to the difference in their data size. In USLCI, the limited number of processes makes it challenging for the model to consistently find truly similar processes. This limited candidate pool potentially compromises the model's performance. In contrast, the larger database likely provides a more diverse set of processes, allowing the model to find more accurate matches and thus perform better.

## DISCUSSION

In this study, we utilized a similarity-based method to discuss the potential data issues when applying ML framework in LCA studies. While it may appear limiting, focusing on one general and widely recognized model allows us to delve into the data-related challenges within the LCI database without adding additional complexity of comparing multiple ML algorithms. Our findings primarily address structural issues within the LCI database, which are not unique to the selected method but instead represent common challenges impacting a variety of ML approaches.

In addition, we set the 5% of missing data as our case scenario as our approach is not intended to replace the collection of primary data for unit processes but rather to offer a complementary solution when small part of primary data is unavailable. Although this percentage may seem minor, it serves as a representative scenario to examine the limitations and robustness of ML models under manageable levels of data absence. Addressing scenarios with higher proportions of missing data is beyond the scope of this study but highlights an important limitation of current ML approaches, which are only applicable to cases with relatively small-scale missingness.

Typically, the parameter *q* is set to a value greater than or equal to 1 to ensure the mathematical properties of a distance metric, such as the triangle inequality, are maintained. However, in our study, we experimented with *q* values in a wide range and the optimal value is less than 1 with meaningful and insightful results. As the LCI data are typically extremely sparse, the value of *q* < 1 effectively emphasizes small differences between components while de-emphasizing larger ones, allowing us to capture subtle similarities between data points that may be overlooked with traditional distance metrics. Despite not adhering to the properties of a metric space, a *q* value less than 1 can serve as a useful dissimilarity measure in specific contexts where capturing local structure is more informative than global distance measures. The use of *q* < 1, though unconventional, can offer advantages in exploratory data analysis. In light of these insights, the results we obtained using *q* < 1 provide valuable contributions to our understanding of the dataset's underlying patterns. Moving forward, we aim to explore additional parameter settings and methodologies to further validate and extend our findings.

Despite several limitations for data-driven solutions for LCI having been identified in this study, we still foresee great potential and aim to provide some suggestions for future research to encourage further development in this field.

- Similar to our method, other existing ML methods also tend to use single/few sources of information from a given database for embedding the training data (e.g., only use the flow values to embed a unit process). It may be feasible to conduct multi-source data fusion using other information to formulate a high-dimension embedding space (e.g., ISIC code, word embedding of the description of a unit process) in future studies to provide more accurate and less biased estimations (alleviate the dominant effects of the single source data). In addition, data scarcity and quality are the top issues that impede ML applications. An incomplete or insufficient inventory may provide additional difficulty in data-driven modeling attempts. Thus, data integration from multiple sources to obtain a larger dataset should be promoted and encouraged in future studies to address the potential data imbalance issues. Meanwhile, building an open-access data-sharing community is also essential to increase the size and diversity of data. In this way, we can foster the improvement of ML models and promote greater collaboration across diverse fields.
- When presenting model performance, it is important to acknowledge the potential variation due to random splits. Reporting performance metrics as averages and providing confidence intervals or standard deviations can help convey the reliability of the results. Techniques such as *k*-fold cross-validation or LOOCV can also be used to provide a more robust evaluation of the model performance and make informed decisions about the suitability of a given ML method for their specific problem. Meanwhile, as any changes in the model training process (e.g., data split and evaluation metrics) could influence the final model performance, there is a needed call for the application of the findability, accessibility, interoperability, reusability (FAIR) principles (Wilkinson et al., 2016) to all of the ML applications in this field, for example, enhance the documentation of the methods to facilitate the reproducibility and reusability of the results.
- Moreover, when handling datasets like the UPR dataset, it is crucial to balance maintaining the “physical meaning” of the data with the application of necessary mathematical transformations. Considering the special characteristics of the UPR dataset, we cannot simply treat the dataset from a purely mathematical perspective. Our study utilized the min–max normalization to eliminate the impact of unit selection variations, which guarantees the consistency of the model outputs. In the future, other ML techniques which are invariant to unit conversion, like random forest, could be applied to meet the special need for the UPR dataset. Additionally, in some cases, we also need to identify and incorporate relevant physical constraints and cannot simply abstract away the “physical meaning” and directly “translate” the physical system to mathematical models (Carranza-Abaid et al., 2020). Physics-informed machine learning offers a promising future direction by integrating physical principles within the ML framework. This approach could enhance model reliability and interpretability, especially in cases of sparse or large proportions of data missing, thus complementing existing modeling frameworks and addressing some of the challenges identified in our study.
- In our current results, we did not consider that the importance of flows may change with respect to different impact categories. For example, a less accurate estimation of heavy metal emissions, given their typically small quantity, may not lead to a considerable difference in the GWP results of a process. However, the corresponding difference in ecotoxicity results may be significant. Therefore, for future studies, this framework may be further integrated with the impact assessment methods to involve weights (such as using the characterization factors for different midpoint categories as weights) in the estimation process.

With further improvement in these mentioned perspectives, data-driven ML techniques may achieve wider applications that further facilitate the construction of better and more robust LCI infrastructures.

## CONCLUSIONS

In this study, we identified key challenges in applying ML methods to estimate missing LCI data using a similarity-based framework with the Ecoinvent 3.1 database. Our analysis reveals that data imbalances, particularly in flow and process availability and magnitude diversity, contribute significantly to model instability and limit reliability. The conventional ML development workflow, including data preprocessing and training practices, often fails to capture the complexities of LCI data. Specifically, the random train–test splits can introduce fluctuation in the model performance and the generalizability of ML models may be hampered by varying database sizes and structures, as demonstrated in tests with the USLCI. To address these issues, future research should focus on improving database integration, enhancing data preparation techniques, and developing adaptable ML frameworks. These efforts are vital for leveraging ML to effectively bridge inventory data gaps and support the broader application of LCA.

## Supplementary Information

This supporting information provides the data from Figure1-7 and S1, Table S1, and Figure S1-S3.

Supporting info item

## Data Availability

The code used to generate the figures of this study can be found at: https://github.com/jitongj/LCA-code. The data used for generating the figures are available upon request.
